# Intestinal-Specific TNFα Overexpression Induces Crohn’s-Like Ileitis in Mice

**DOI:** 10.1371/journal.pone.0072594

**Published:** 2013-08-20

**Authors:** Giorgos Bamias, Mohamed I. Dahman, Kristen O. Arseneau, Mitchell Guanzon, Dennis Gruska, Theresa T. Pizarro, Fabio Cominelli

**Affiliations:** 1 1st Department of Gastroenterology, Ethnikon & Kapodistriakon University of Athens, Laikon Hospital, Athens, Greece; 2 Department of Surgery, Mercy Health System, Fairfield, Ohio, United States of America; 3 Departments of Medicine and Pathology, Case Western Reserve University School of Medicine, Cleveland, Ohio, United States of America; 4 Digestive Health Research Center, Case Western Reserve University, Cleveland, Ohio, United States of America; McMaster University, Canada

## Abstract

**Background and Aim:**

Human and animal studies have clearly established tumor necrosis factor (TNF)α as an important mediator of Crohn’s disease pathogenesis. However, whether systemic or only local TNFα overproduction is required for the development of chronic intestinal inflammation and Crohn’s disease remains unclear. The aim of this study was to assess the contribution of intestinal epithelial-derived TNFα to the development of murine Crohn’s-like ileitis.

**Methods:**

We adapted the well-established TNF^∆ARE/+^ mouse model of Crohn’s disease (which systemically overexpresses TNFα) to generate a homozygous mutant strain that overexpress TNFα only within the intestinal epithelium. Intestinal-specific TNF^i∆ARE/i∆ARE^ mice were examined for histopathological signs of gut inflammation and extraintestinal manifestations of Crohn’s disease. The mucosal immune phenotype was characterized, and the contribution of specific lymphocyte populations to the pathogenesis of TNF^i∆ARE/i∆ARE^ ileitis was assessed.

**Results:**

TNF^i∆ARE/i∆ARE^ mice had increased mucosal and systemic TNFα levels compared to wild-type controls (P<0.001), as well as severe chronic ileitis with increased neutrophil infiltration and villous distortion, but no extraintestinal manifestations (P<0.001 vs. wild-type controls). The gut mucosal lymphocytic compartment was also expanded in TNF^i∆ARE/i∆ARE^ mice (P<0.05), consisting of activated CD69^+^ and CD4^+^CD62L^-^ lymphocytes (P<0.05). FasL expression was significantly elevated in the mesenteric lymph nodes of TNF^i∆ARE/i∆ARE^ mice (P<0.05). Adoptive transfer of mucosal TNF^i∆ARE/i∆ARE^ lymphocytes resulted in ileitis in immunologically naïve severe combined immunodeficiency recipients (P<0.05 vs. wild-type controls), indicating an effector phenotype that was associated with increased production of both Th1 (IFNγ) and Th2 (IL-5, IL-13) cytokines.

**Conclusion:**

Intestinal epithelial-derived TNFα is sufficient for the induction of Crohn’s-like ileitis, but not for the occurrence of extraintestinal manifestations, in TNF^i∆ARE/i∆ARE^ mice. These effects were associated with generation of effector lymphocytes within the intestinal mucosa and dysregulated apoptosis. Thus, targeted intestinal blockade of TNFα may provide an effective means to neutralize gut-derived TNFα with reduced side effects.

## Introduction

Ulcerative colitis (UC) and Crohn’s disease (CD) are chronic intestinal disorders, collectively referred to as inflammatory bowel disease (IBD). Despite significant progress in recent years, the exact cause of these diseases remains unclear. According to the most widely accepted hypothesis, IBD develops in genetically predisposed individuals due to a dysregulated immune response against constituents of the commensal flora, under the influence of undefined environmental triggers [1]. As mediators of innate and adaptive immune responses, several intestinal cytokines and/or their cognate receptors have been implicated in IBD pathogenesis [2].

TNFα is a proinflammatory cytokine and the prototypic member of the TNF superfamily of proteins, a large group of molecules that are associated with most aspects of immunity [3]. The pivotal role of TNFα in the pathogenesis of IBD has been clearly established through several lines of evidence. First, the expression of TNFα is elevated in affected mucosal areas of patients with IBD, both at the mRNA and protein levels [4,5]. Likewise, in animal models of intestinal inflammation, TNFα is significantly upregulated in the presence of active disease [6]. Moreover, mice that have been genetically manipulated to systemically overexpress TNFα (TNF^ΔARE/+^ mice) develop chronic ileitis with marked similarities to Crohn’s ileitis in humans, as well as extraintestinal CD manifestations, such as inflammatory arthritis [7]. In addition, inhibition of TNFα activity results in amelioration of experimental intestinal inflammation in mice [6,8]. However, the strongest evidence by far comes from clinical trials in humans that show neutralizing monoclonal antibodies against TNFα to be highly effective at treating refractory and/or fistulizing CD and UC [9,10]. Application of these anti-TNF drugs to clinical practice has greatly benefited IBD patients, and provides direct evidence for the involvement of TNFα in the pathogenesis of IBD [9–13]. A greater understanding of the relationship between TNFα overexpression and chronic intestinal inflammation may result in new classes of anti-TNF therapies that more directly target TNFα overexpression and its pathogenic source, thereby limiting the risk of drug-induced toxicities.

TNF^∆ARE^ mice carry a genetic deletion in the AU-rich elements (ARE) contained within the 3’ untranslated region of their TNFα mRNA transcripts. This deletion leads to enhanced TNFα mRNA stability and systemic over-production of the translated protein [7,14,15]. Heterozygous TNF^∆ARE/+^ mice display an inflammatory phenotype that is most prominently expressed in the joints and small intestine with development of arthritis and ileitis, respectively, and offers the unique opportunity to study not only TNFα-mediated inflammatory mechanisms in the small intestine, but also the pathogenesis of extraintestinal manifestations of CD. The deletion in TNF^∆ARE/+^ mice was introduced in embryonic stem cells, causing the increase in TNFα mRNA stabilization to occur globally. Therefore, it is difficult to dissect the contributory roles of individual cell types, such as immunocytes, epithelial cells, and mesenchymal cells, to disease pathogenesis in this mouse strain. In addition, it is not known whether systemic or localized TNFα-mediated immunological effects are required to generate the chronic intestinal inflammation characteristic of CD.

In order to isolate the effects of TNFα in the intestinal microenvironment, we generated mice that carry the deletion in the TNFα ARE and express murine TNFα under the promoter of the intestinal fatty acid binding protein (I-FABP) (TNF^i∆ARE/i∆ARE^ mice). As the I-FABP gene is expressed on intestinal epithelial cells (IEC) s exclusively, this approach allowed us to study the local effects of TNFα overexpression, specifically derived from the intestinal epithelium. In the present study, we performed a detailed histopathological and immunological characterization of this novel TNF^i∆ARE/i∆ARE^ strain, and were able to demonstrate increased mucosal expression of TNFα protein. However, epithelial-derived overexpression of TNFα was only sufficient to induce mucosal inflammation, with no signs of the extraintestinal inflammation observed in these mice. Intestinal inflammation in TNF^i∆ARE/i∆ARE^ mice was mediated through the generation of effector CD4^+^ lymphocytes, which expressed markers of activation, adoptively transferred ileitis to immunologically naïve recipients, and secreted pro-inflammatory cytokines of both Th1 and Th2 immunophenotypes.

## Materials and Methods

### Animals

Generation of TNF^ΔARE/ΔARE^, TNF^ΔARE/+^, and TNF^ΔAREneo/neo^ mice have been previously described [7]. Intestinal-specific TNF^i∆ARE/i∆ARE^ mice were generated by crossing TNF^ΔAREneo/neo^ mice, which carry *Tnf* ^ΔAREneo^ alleles that have a LoxP-flanked neomycin (neo) cassette inserted next to the *tnf* ^ΔARE^ mutation such that the *tnf* ^ΔARE^ mutation can be activated upon cre-mediated deletion of the neo cassette, with *Fabpl* ^*4× at −132*^ */Cre* transgenic mice that express Cre recombinase under the control of the intestinal-specific Fabp (I-FABP) promoter (*Fabpl* ^*4× at −132*^ */Cre* transgenic mice were kindly provided by Dr. Jeffrey Gordon of Washington University, St. Louis). [16]. The resulting intestinal-specific TNF^i∆ARE/i∆ARE^ mice express the *tnf* ^ΔARE^ mutation and overproduce TNFα only within the intestinal epithelium. All mice were evaluated at 16-20 weeks of age.

All mice were bred and maintained at the Case Western Reserve University. These included TNF^+/+^ wild-type (wt) mice, TNF^ΔAREneo^, TNF^ΔARE/+^, TNF^ΔARE/ΔARE^, and intestinal-specific TNF^i∆ARE/i∆ARE^, as well as *Fabpl^4× at −132^/Cre* transgenic and SCID mice (C3H/HeJ background). All procedures were approved by the Case Western Reserve University Institutional Animal Care and Use Committee.

### Histology

Histologic evaluation was performed in H&E-stained sections of intestinal tissues fixed in 10% formalin solution. Quantification of intestinal lesions was done in a blinded fashion by a single pathologist, using a validated scoring system, as previously described [17]. In brief, histologic indices were evaluated for (1) active inflammation (infiltration with neutrophils), (2) chronic inflammation (lymphocytes, plasma cells, and macrophages in the mucosa and submucosa), and (3) villus distortion (flattening and/or widening of normal villus architecture). For each index a score ranging from 0 (normal histology) to 3 (maximum severity of histologic changes) was applied. The sum of all 3 individual components was expressed as the total inflammatory score.

### Cell Isolation

Following aseptic removal, mesenteric lymph nodes (MLN) s were gently pressed against a 100-µm cell strainer to obtain single-cell suspensions. For lamina propria (LP) mononuclear cell isolation, intestines were removed, washed with cold PBS, and cut into 2- to 5-mm pieces. To remove epithelial cells and debris, intestinal pieces were placed in Hanks’ balanced salt solution with 15 mmol/L HEPES and 1 mmol/L ethylenediaminetetraacetic acid, and vortexed at room temperature until the incubating solution became clear. Tissues were then placed into digestion solution (RPMI 1640, with 10% fetal bovine serum, 15 mmol/L HEPES, 1% penicillin/streptomycin, and 100–200 U/mL collagenase VIII (Sigma Immunochemicals, St. Louis, MO). Following incubation at 37°C for 60 minutes, lymphocyte-enriched populations were isolated at the 40%/100% interface of a discontinuous Percoll gradient.

### Cell Sorting

CD4^+^ enriched populations were isolated from single cell suspensions of MLNs from TNF^i∆ARE/i∆ARE^ or wt control mice after incubation with anti-CD4-bound magnetic beads. Positive selection into the respective populations was performed by use of a magnetic cell-sorting system (Miltenyi Biotec, Auburn, CA). Purity of sorted cells for CD4 was 95%.

### Cell Culture

Single cell suspensions from MLNs were cultured in 96-well round-bottom plates at 10^6^ cells/mL of complete medium (RPMI 1640 with 10% fetal bovine serum, 2 mmol/L L-glutamine, and 1% penicillin/streptomycin). Cultures were performed in unstimulated conditions or under stimulation with immobilized anti-CD3 mAb (10 g/mL; BD Biosciences PharMingen, San Diego, CA). After 48 hours, the cells were harvested, centrifuged, and pellets stored at 80°C until further testing.

### Flow Cytometry

Single-cell suspensions from MLNs or the LP were incubated with the appropriate combinations of fluorochrome-tagged monoclonal antibodies against CD4, CD69, CD25, CD62L, Fas, and FasL (BD Biosciences PharMingen) and fixed in 1% paraformaldehyde. Three-color flow cytometry was performed on a FACS Calibur System ((BD Biosciences Immunocytometry Systems, San Jose, CA) and the percentage of cells expressing surface markers, as well as the intensity of expression, was determined.

### Adoptive Cell Transfer

Purified CD4^+^ cells (1x10^6^) obtained from the MLNs of TNF^i∆ARE/i∆ARE^ and wt mice (14-18 weeks of age) were adoptively transferred by intraperitoneal injection into MHC-matched SCID mice (6-8 weeks of age). Recipient mice were euthanized 8 weeks after the transfer. Small and large intestines were excised for histological assessment of inflammation and MLN cells cultured for cytokine secretion measurements.

### Cytometric Bead Array

For the concomitant measurement of IL-2, TNFα, IFNγ, IL-4, and IL-5 protein levels in cell culture supernatants, the mouse Th1/Th2 Cytometric Bead Array (BD Biosciences PharMingen, San Diego, CA) was used, according to the manufacturer’s instructions. Samples were analyzed on a FACS Calibur (BD Biosciences Immunocytometry Systems, San Jose, CA) with the use of BD CBA software (BD Biosciences PharMingen).

### ELISA

The concentration of TNFα protein in tissue homogenates and mouse sera was measured by a commercially available enzyme-linked immunosorbent assay (R&D Systems, Minneapolis, MN). In tissue homogenates, values were expressed as pg of TNF per mg of total protein content, which was measured by the Bio-Rad Protein Assay (Bio-Rad Laboratories, USA).

### Statistical Analysis

Statistical analyses were performed using a 2-sided Student *t* test or nonparametric Wilcoxon Rank Sum test, depending on the size of the samples to be compared. An α-level of 0.05 was considered significant (P<0.05).

## Results

### Increased mucosal expression and systemic circulation of TNFα in TNF^i∆ARE/i∆ARE^ mice

**Figure 1:**
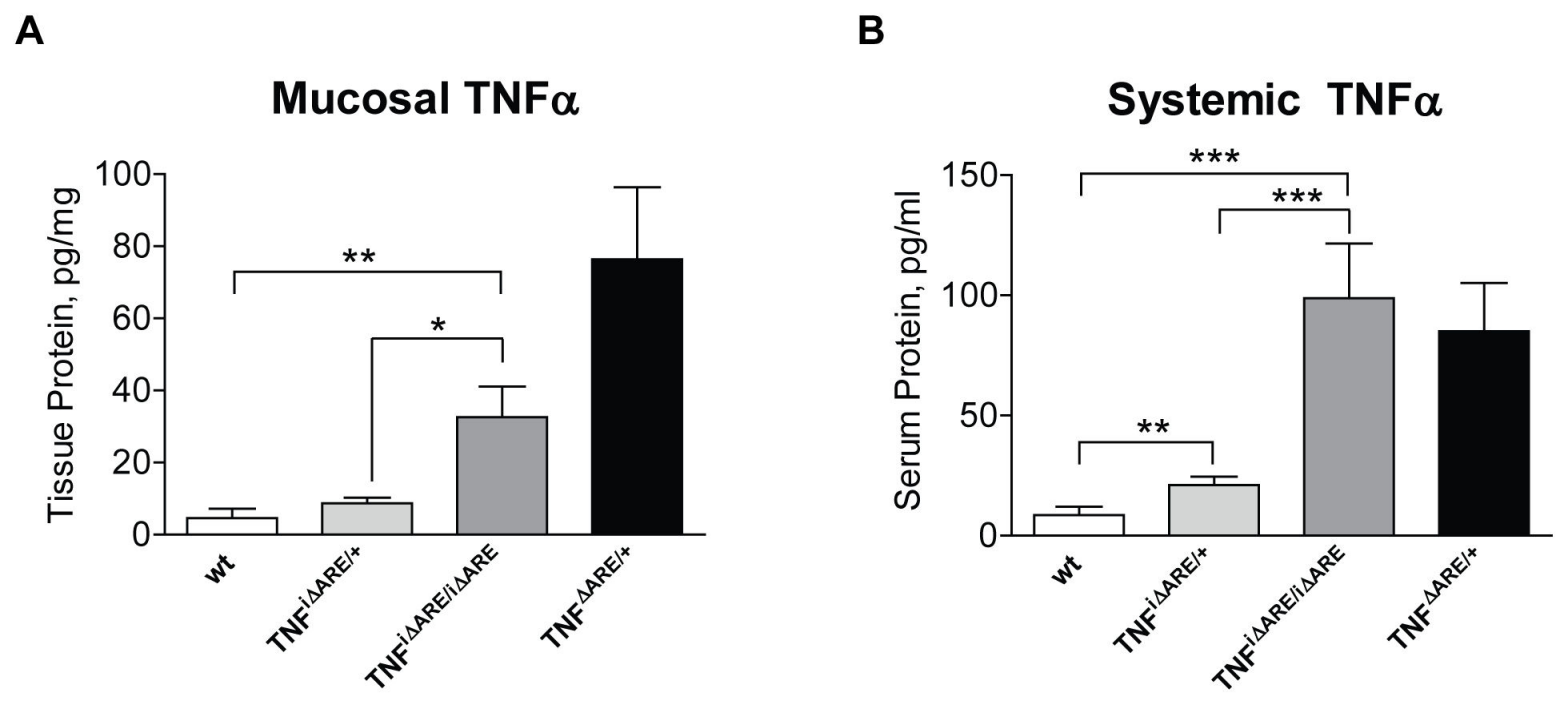
Mucosal and systemic expression of TNFα protein is elevated in TNF^i∆ARE/i∆ARE^ mice. Intestinal epithelial-specific overexpression of TNFα was induced by expressing the *tnf* ^ΔARE^ mutation under the promoter of the intestinal-specific I-FABP gene, which is expressed primarily by IECs. (A) Pieces of small intestinal tissue were homogenized at the time of animal sacrifice, and the concentration of TNFα protein was measured by ELISA. Values are expressed as pg of TNFα per mg of total protein content, which was measured by a Bradford Protein Assay. (B) Blood was drawn from mice by cardiac puncture at the time of sacrifice. Serum was collected and stored until use. The concentration of TNFα protein was measured by ELISA. Murine ileal tissue and sera were analyzed individually. A significant increase in the total protein content in both small intestinal tissue homogenates and murine sera was seen in homozygous TNF^i∆ARE/i∆ARE^ mice (n=11), as compared with either heterozygous TNF^i∆ARE/+^, (n=10) or wt mice (combined wt for TNF^i∆ARE/i∆ARE^ and systemic TNF^ΔARE/+^, n=16). Mice with systemic overexpression of TNFα were included in the analysis, serving as positive controls (TNF^ΔARE/+^ mice, n=6). Graphs represent mean values ± SEM for each experimental group of mice. *P<0.05, ** < P<0.01, *P<0.001.

To study the contribution of epithelial-derived TNFα overexpression to the mechanisms that underlie mucosal inflammation, we generated mice that express mouse TNFα under the promoter of the I-FABP gene, which is primarily expressed on small IECs (TNF^i∆ARE/i∆ARE^ mice). To validate our experimental system, we measured TNFα protein levels in homogenates of small intestinal tissue from TNF^i∆ARE/i∆ARE^ mice in comparison to wt controls. Our results showed that TNF^i∆ARE/i∆ARE^ mice had significantly elevated mucosal TNFα protein levels in comparison to wt and heterozygous TNF^i∆ARE/+^ mice (mean ileal TNFα level: TNF^i∆ARE/i∆ARE^ = 32.64 ± 8.50 pg/mg tissue; TNF^i∆ARE/+^ mice = 8.73 ± 1.65 pg/mg tissue, P=0.01 vs. homozygotes; wt = 4.62 ± 2.56 pg/mg tissue, P=0.02 vs. homozygotes, Figure 1A). As expected, we detected highly elevated concentrations of mucosal TNFα protein in systemic TNF^ΔARE/+^ mice (76.39 ± 20.04 pg/mg tissue), which served as positive controls, as they have been shown to develop TNF-mediated intestinal inflammation [7].

Next, we examined whether epithelial-specific TNFα overexpression was also associated with increased levels of TNFα in the systemic circulation (Figure 1B). We showed that TNF^i∆ARE/i∆ARE^ mice had significantly higher circulating levels of TNFα when compared with wt control mice (TNF^i∆ARE/i∆ARE^: 98.8 ± 22.7 pg/ml vs. wt: 8.5 ± 3.5 pg/ml, P<0.005). In fact, TNFα levels were comparable between TNF^i∆ARE/i∆ARE^ and systemic TNF^ΔARE/+^ mice (85.1 ± 20.1 pg/ml, NS). A gene dose-effect was observed as heterozygous TNF^i∆ARE/+^ mice had intermediate levels of serum TNFα (TNF^i∆ARE/+^: 21.0 ± 3.5 pg/ml, vs. homozygotes P<0.005, or wt P<0.05). Taken together, these results confirm that successful overexpression of TNFα at the mucosal level in our genetically-engineered TNF^i∆ARE/i∆ARE^ mouse strain also leads to elevated systemic levels, likely due to TNFα spillover from the intestinal epithelium.

### Intestinal-specific TNF^i∆ARE/i∆ARE^ mice display severe ileitis but lack extraintestinal manifestations

**Figure 2:**
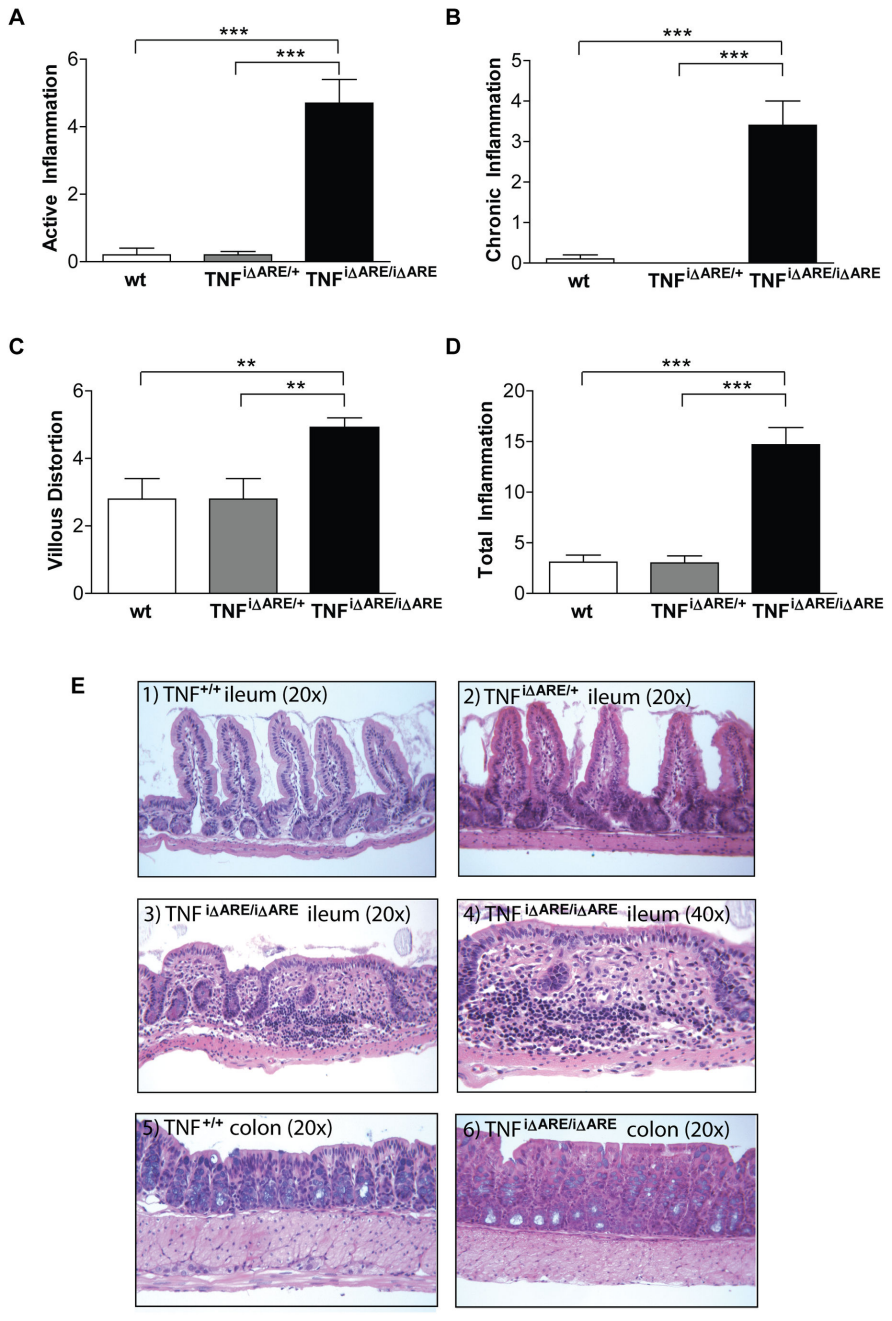
Histopathological features of TNF^i∆ARE/i∆ARE^ mice. Histological assessment of inflammation in the terminal ileum was done using a validated scoring system. Indices were calculated for (A) villous distortion, (B) active inflammation (neutrophil infiltration), and (C) chronic inflammation (mononuclear cell infiltration). (D) The total inflammatory score represents the sum of all 3 individual indices. Values for all indices were significantly elevated in homozygous TNF^i∆ARE/i∆ARE^ mice (n=11), whereas no inflammatory changes were detected in heterozygous TNF^i∆ARE/+^ mice (n=10) or wt mice (n=10); all mice were evaluated at 16-20 weeks of age. Graphs represent mean values ± SEM for each group of mice. *P<0.05, ** < P<0.01, *P<0.001. (E) Representative photomicrographs of H&E stained sections from wt TNF^+/+^, TNF^i∆ARE/+^, and TNF^i∆ARE/i∆ARE^ mice. 1) wt TNF^+/+^ ileum showing normal villous architecture with no blunting or evidence of acute or chronic inflammatory infiltration, 20x. (2) TNF^i∆ARE/+^ ileum showing mild villous blunting with no significant inflammation, 20x. (3) TNF^i∆ARE/i∆ARE^ ileum showing evidence of chronic ileitis with villous blunting; acute and chronic inflammatory infiltration is seen within the LP and extending into the submucosa, 20x. (4) Higher magnification of ileum histology from a TNF^i∆ARE/i∆ARE^ mouse showing evidence of marked inflammatory infiltration and distortion of normal villous architecture and crypts, 40x. (5) Colon histology of wt TNF^+/+^ mice showing no evidence of colonic inflammation, 20x. (6) Colon histology of a TNF^i∆ARE/i∆ARE^ mouse showing normal colonic histology with only a few lymphocytes infiltrating the LP.

We next studied whether the intestinal epithelial-derived elevation in mucosal and systemic TNFα resulted in a specific disease phenotype in TNF^i∆ARE/i∆ARE^ mice, using systemic TNF^ΔARE/+^ mice as the reference model since these mice display an arthritis/ileitis inflammatory phenotype. We first examined terminal ileal sections for the presence of intestinal inflammation, which was quantified using a well-validated histological scoring system for villous blunting, and active, chronic, and total inflammation [17]. Histological evaluation revealed severe inflammatory changes in the ilea of TNF^i∆ARE/i∆ARE^ mice, but not in heterozygous TNF^i∆ARE/+^ mice or wt littermates (Figure 2). Pathological changes included dense infiltration of the LP by acute (polymorphonuclear), but mainly chronic (lymphocytes, macrophages), inflammatory cells. This in turn led to disruption of the mucosal architecture with prominent villous distortion that was characterized by widening and decreased number of villi. In particular, the active inflammatory index, which correlates to the presence of neutrophils, was 4.7 ± 0.7 in TNF^i∆ARE/i∆ARE^ mice, 0.2 ± 0.1 in TNF^i∆ARE/+^ mice, and 0.2 ± 0.2 in wt mice (P<0.0001 vs. homozygotes for both comparisons, Figure 2A). The chronic inflammatory index, which reflects lymphocytic infiltration, was 3.4 ± 0.6 in TNF^i∆ARE/i∆ARE^ mice, whereas no such infiltration was noted in the two other groups (P<0.0001 vs. homozygotes for both comparisons, Figure 2B). Finally, the villous distortion index, which correlates to the severity of epithelial changes, was also significantly higher in TNF^i∆ARE/i∆ARE^ mice (4.9 ± 0.3) than in TNF^i∆ARE/+^ (2.8 ± 0.6, P<0.01) or wt mice (2.8 ± 0.6, P<0.01, Figure 2C). In all, mean total inflammatory scores were 14.7 ± 1.7 in TNF^i∆ARE/i∆ARE^ mice, 3.0 ± 0.7 in TNF^i∆ARE/+^ mice (P<0.0001) and 3.1 ± 0.7 in wt mice (P<0.0001, Figure 2D). As the I-FABP promoter is also expressed in the proximal colon, we also evaluated TNF^i∆ARE/i∆ARE^ mice for the presence of colitis. Low-grade inflammatory cell infiltration with both active and chronic components was seen in the colons of TNF^i∆ARE/i∆ARE^ mice, but was not present in either TNF^i∆ARE/+^ or wt mice (mean total colitis scores: TNF^i∆ARE/i∆ARE^ = 3.3 ± 1.0; TNF^i∆ARE/+^ = 0.6 ± 0.4, P<0.05 vs. homozygotes; wt = 0.4 ± 0.3, P<0.05 vs. homozygotes). Conversely, no detectable inflammation was observed in other parts of the small intestine, including the jejunum.

In previous studies, it has been shown that the systemic counterpart of our model, i.e. TNF^ΔARE/+^ mice, display an inflammatory arthritis phenotype in addition to ileitis [7]. Therefore, we sought to determine whether extraintestinal Crohn’s-like manifestations were also present in TNF^i∆ARE/i∆ARE^ mice, especially since we observed increased systemic levels of TNFα in these mice. Our studies clearly demonstrated that TNF^i∆ARE/i∆ARE^ mice did not develop the inflammatory arthritis that is characteristic of the systemic TNF^ΔARE/+^ strain. In addition, no histological signs of joint injury were detected in TNF^i∆ARE/i∆ARE^ mice (data not shown). Taken together, these studies indicate that intestinal epithelial-derived overexpression of TNFα is sufficient for the induction of chronic intestinal inflammation. However, local TNFα overexpression in the joints is required for extraintestinal arthritic lesions to manifest themselves.

### Increased expression of activation and apoptotic markers on gut mucosal lymphocytes from TNF^i∆ARE/i∆ARE^ mice

To understand the mechanisms through which increased intestinal epithelial expression of TNFα leads to intestinal inflammation, we next examined the composition and activation status of the cellular compartment within the MLNs and small intestinal LP of TNF^i∆ARE/i∆ARE^ mice, focusing primarily on CD4^+^ lymphocytes. Our studies clearly demonstrate a significant increase in the proportion of MLN CD4^+^ cells that expressed various markers of activation. In particular, the percentage of CD4^+^ cells expressing CD69 was increased from 14.32 ± 1.67% in wt mice to 33.74 ± 1.54% in TNF^i∆ARE/i∆ARE^ mice (P<0.05, Table 1), and the percentage of CD4^+^ cells lacking CD62L expression increased from 11.19 ± 0.93% to 37.17 ± 0.44% (P<0.05). Finally, the percentage of CD4^+^ cells expressing CD25 was 11.57 ± 0.70% in wt mice and 25.04 ± 1.22% in TNF^i∆ARE/i∆ARE^ mice (P<0.05). In all cases, percentages of activated CD4^+^ cells were comparable between intestinal and systemic TNF^ΔARE^ mice. In addition, when we looked at freshly isolated LP mononuclear cells from TNF^i∆ARE/i∆ARE^ mice, we were able to demonstrate very large numbers of activated CD4^+^ lymphocytes (CD4^+^CD69^+^: 74.70 ± 1.70%, CD4^+^CD62L^-^: 92.13 ± 0.85%, and CD4^+^CD25^+^: 48.72 ± 0.96%). We also measured the number of CD4^-^ cells for the expression of activation markers. Our results showed that the number of CD69^+^ cells in this population was significantly elevated in MLNs from TNF^i∆ARE/i∆ARE^ mice (27.91 ± 3.00%) as compared to wt littermates (9.65 ± 1.59%, P<0.05) (data not shown). However, no significant differences were observed between TNF^i∆ARE/i∆ARE^ mice and wt mice regarding the percentages of CD25^+^ or CD62L^-^ cells within the CD4^-^population (data not shown).

**Table 1:** Percent of total CD4^+^ MLN lymphocytes expressing cell surface activation markers.

Activation Marker	wt TNF^+/+^ MLN	TNF^ΔARE/+^ MLN	TNF^iΔARE/ iΔARE^ MLN	TNF^iΔARE/ iΔARE^ LP
		(P-value vs. wt)	(P-value vs. wt)	(P-value vs. wt)
CD69^+^	14.32 ± 1.67	32.05 ± 5.63	33.74 ± 1.54	74.70 ± 1.70
		(0.043)	(0.021)	(0.021)
CD62L^-^	11.19 ± 0.93	28.18 ± 4.11	37.17 ± 0.44	92.13 ± 0.85
		(0.021)	(0.021)	(0.021)
CD25^+^	11.57 ± 0.70	29.66 ± 2.41	25.04 ± 1.22	48.72 ± 0.96
		(0.021)	(0.021)	(0.021)

Immunophenotypic characterization of mucosal lymphocytes from TNF^i∆ARE/i∆ARE^ mice with ileitis. Single cell suspensions were obtained from MLNs and small intestinal LP. The expression of various surface markers was analyzed by flow cytometry. Fluorochrome-tagged monoclonal antibodies against CD4, CD69, CD25, and CD62L were utilized for identification of the respective populations. Multiple color flow cytometry was performed on a FACS calibur system to determine the percentage of cells expressing surface markers and the intensity of expression. A significant increase was observed in the proportion of mucosal (MLN and LP) CD4^+^ cells that expressed various markers of activation. The percentages of CD4^+^ cells expressing CD69 or CD25, or lacking expression of CD62L, were significantly elevated in the MLNs of TNF^i∆ARE/i∆ARE^ mice in comparison to wt controls (P<0.05), whereas no differences were seen between intestinal TNF^i∆ARE/i∆ARE^ and systemic TNF^ΔARE/+^ mice. The largest percentages of CD4^+^ lymphocytes expressing markers of activation were detected in freshly isolate LP mononuclear cells from TNF^i∆ARE/i∆ARE^ mice. Mice were processed individually. Three separate experiments with 4 mice per group gave similar results. Data are represented as mean values ± SEM for each group of mice.

**Figure 3:**
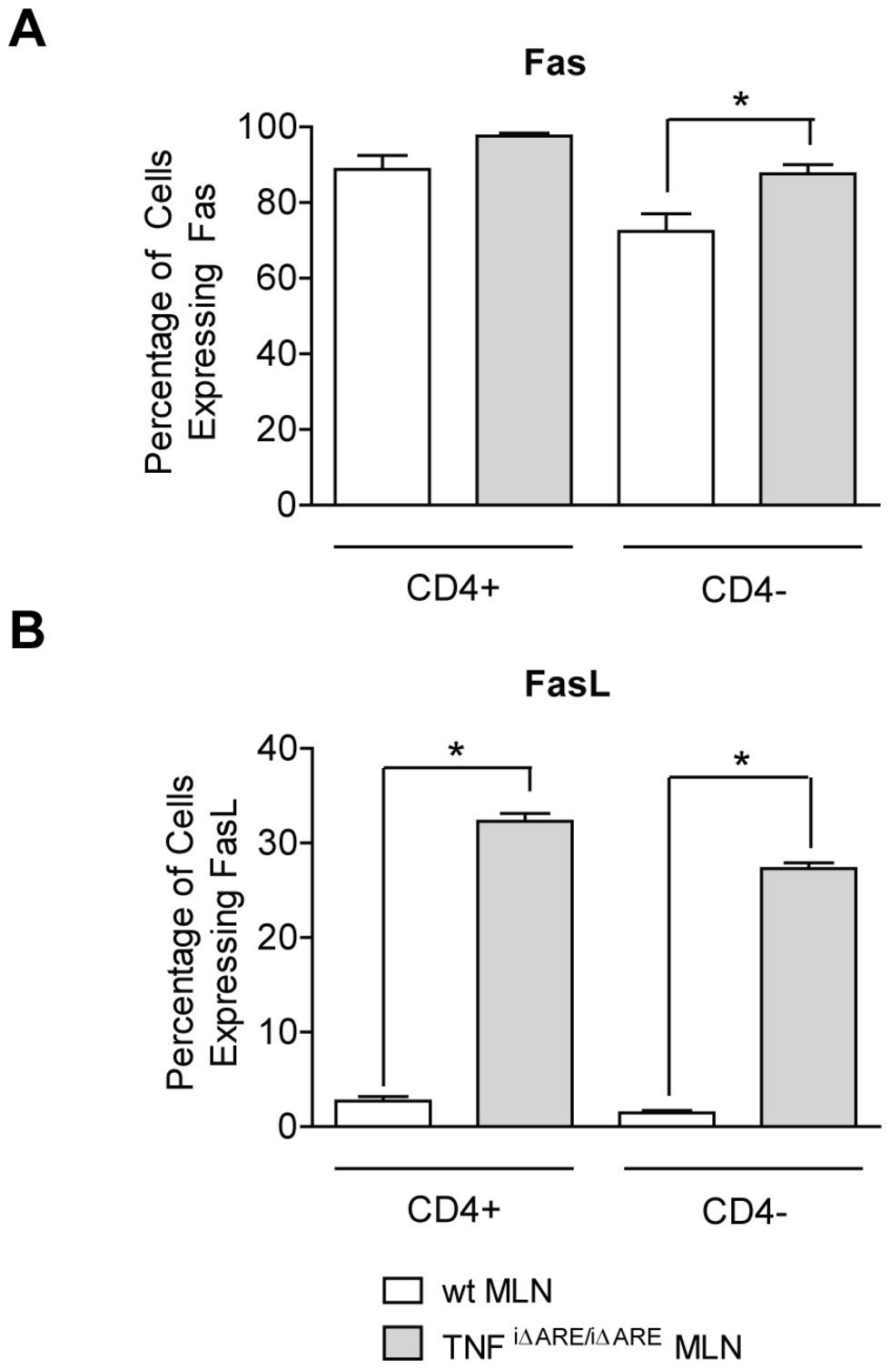
Increased apoptosis of lymphocytes in the MLNs of TNF^i∆ARE/i∆ARE^ mice. Single cell suspensions were obtained from MLNs, and the expression of various surface markers was analyzed by flow cytometry. Fluorochrome-tagged monoclonal antibodies against CD4, Fas, and FasL were utilized for identification of the respective populations. Multiple color flow cytometry was performed on a FACS calibur system to determine the percentage of cells expressing surface markers and the intensity of expression. The vast majority of MLN cells stained positive for Fas and no significant differences detected between wt and TNF^i∆ARE/i∆ARE^ mice. Conversely, expression of FasL was significantly elevated in MLN cells from TNF^i∆ARE/i∆ARE^ mice as compared to wt controls. This difference remained significant when expression of FasL was examined separately in the CD4^+^ or CD4^-^ populations. Mice were processed individually. Three separate experiments with 3-4 mice per group gave similar results, and one representative experiment is shown. Graphs represent mean values ± SEM for each group of experimental mice. *P<0.05, ** < P<0.01, *P<0.001.

Since apoptotic pathways play an important role in the regulation of inflammatory cell removal and are mediated by TNFα, we also examined the expression of the apoptotic receptor-ligand pair, Fas/FasL, on cells isolated from the MLNs of TNF^i∆ARE/i∆ARE^ mice. Our studies show that the vast majority of MLN cells stained positive for Fas, with no significant differences detected between wt and TNF^i∆ARE/i∆ARE^ mice in either total or CD4^+^ and CD4^-^ cell populations (Figure 3). In sharp contrast, expression of FasL was significantly elevated in MLN cells from TNF^i∆ARE/i∆ARE^ mice compared to wt controls. This difference was present when we compared the total number of FasL^+^ cells (TNF^i∆ARE/i∆ARE^: 29.12 ± 0.59% vs. wt: 1.89 ± 0.37%, P<0.05), the number of CD4^+^/FasL^+^ cells (TNF^i∆ARE/i∆ARE^: 33.06 ± 1.24% vs. wt: 2.71 ± 0.46%, P<0.05), or the number of CD4^-^/FasL^+^ cells (TNF^i∆ARE/i∆ARE^: 28.24 ± 0.49% vs. wt: 1.47 ± 0.26%, P<0.05). Taken together, these results show that intestinal epithelial-specific overexpression of TNFα results in enrichment of the mucosal immune compartment with activated lymphocytes and indicate that effector lymphocytic pathways are upregulated in TNF^i∆ARE/i∆ARE^ mice. In addition, the presence of a significantly expanded population of FasL-expressing cells points to the presence of dysregulated apoptotic pathways in these mice.

### Mucosal CD4^+^ lymphocytes from TNF^i∆ARE/i∆ARE^ mice adoptively transfer ileitis to immunocompromised SCID recipients by inducing a mixed Th1/Th2 response

**Figure 4:**
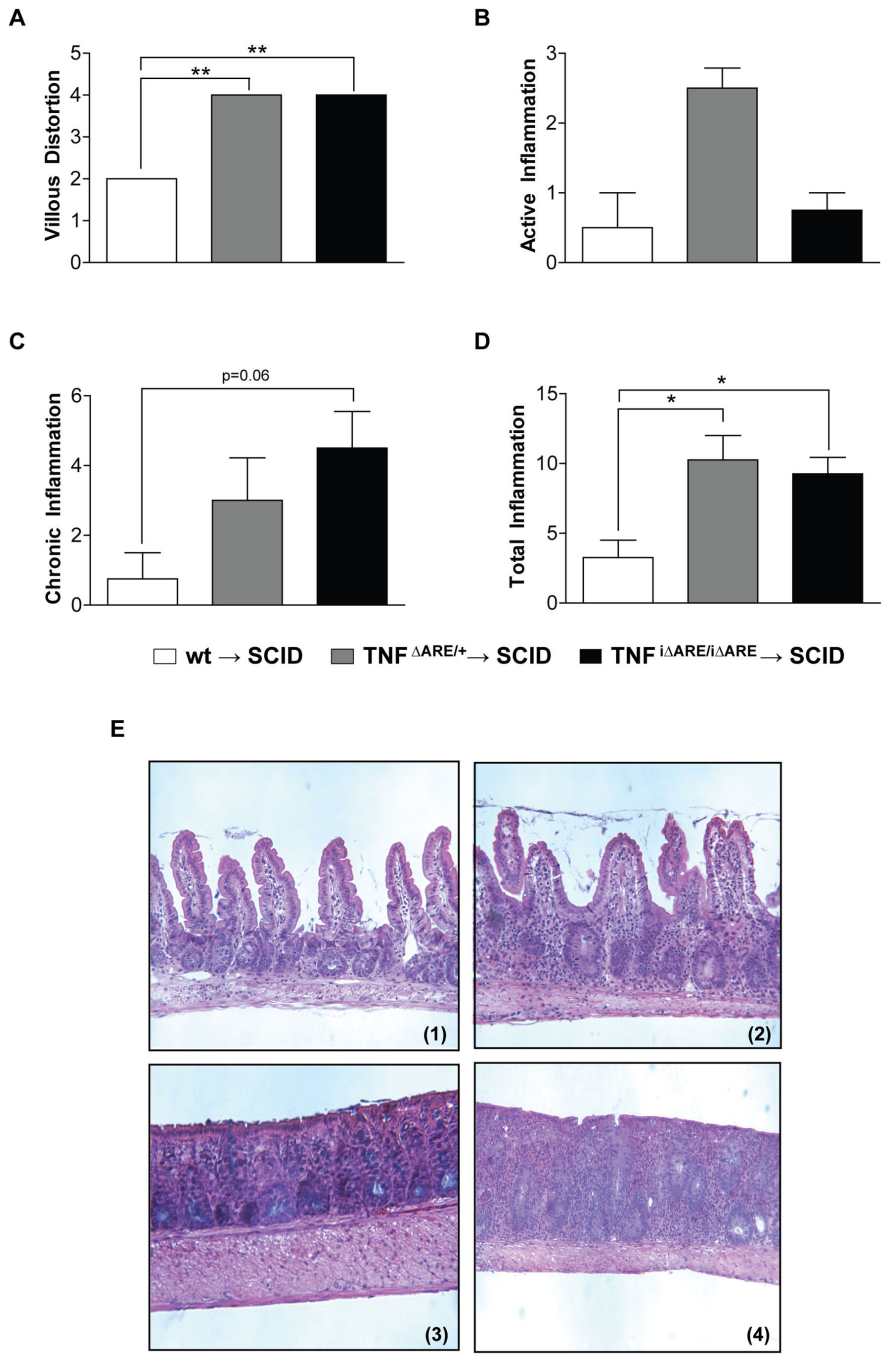
Adoptive transfer of CD4^+^ MLN cells from TNF^i∆ARE/i∆ARE^ mice into SCID recipients. CD4^+^ enriched (purity >95%) populations were obtained from single cell suspensions of MLNs from TNF^i∆ARE/i∆ARE^ via positive selection by use of a magnetic cell-sorting system. Donor cells (1x10^6^) were injected intraperitoneally into MHC-matched 6-8-wk-old SCID mice. Recipient mice were euthanized 8 weeks after the transfer. Histological assessment of inflammation in the terminal ileum was done by a validated scoring system. Indices were calculated for (A) villous distortion, (B) active inflammation (neutrophil infiltration), and (C) chronic inflammation (mononuclear cell infiltration). (D) The total inflammatory score represents the sum of all 3 individual indices. Total inflammation was significantly elevated in TNF^i∆ARE/i∆ARE^ → SCID mice (n=4) and TNF^∆ARE/+^ → SCID mice (n=8), compared to wt TNF^+/+^ → SCID (n=4). Graphs represent mean values ± SEM for each group of mice. *P<0.05, ** < P<0.01, *P<0.001. (E) Representative photomicrographs of H&E stained sections from recipient SCID mice displaying the characteristics of adoptively transferred TNF^i∆ARE/i∆ARE^ → SCID ileitis. (1). Representative wt → SCID mouse showing normal histological appearance of the terminal ileum. (2). Representative TNF^i∆ARE/i∆ARE^ → SCID mouse showing a mixed acute and chronic inflammatory infiltrate with moderate ileitis. Three separate experiments using 4-8 mice per group were performed and gave similar results, and one representative experiment is shown.

We next tested the pathogenic potential of CD4^+^ lymphocytes from the MLNs of TNF^i∆ARE/i∆ARE^ mice, since these mice had increased numbers of activated CD4^+^ cells within their intestinal mucosa, by assessing the ability of these MLN CD4^+^ cells to induce intestinal inflammation upon adoptive transfer to genetically immunocompromised SCID recipient mice. Our results clearly demonstrate the ability of CD4^+^ cells from TNF^i∆ARE/i∆ARE^ mice, but not from wt mice, to induce severe ileitis when transferred to SCID recipients. The histological characteristics of the adoptively transferred ileitis closely resembled those of spontaneous TNF^i∆ARE/i∆ARE^ ileitis (Figure 4A–D). Using our validated histological scoring system, we were able to detect significantly more villous distortion and total inflammation in TNF^i∆ARE/i∆ARE^ → SCID mice compared to wt → SCID mice, and a strong trend towards more chronic inflammation as well (villous distortion index: 4.00 ± 0.00 vs. 2.00 ± 0.00, P<0.001; chronic inflammatory index: 4.5 ± 1.05 vs. 0.75 ± 0.75, P=0.06; total inflammatory score: 9.25 ± 1.19 vs. 3.25 ± 1.25, P<0.05). We observed no difference in the development of intestinal inflammation between SCID mice receiving CD4^+^ MLN cells from intestinal-specific TNF^i∆ARE/i∆ARE^ mice versus systemic TNF^ΔARE/+^ mice (Figure 4 A–E).

**Figure 5:**
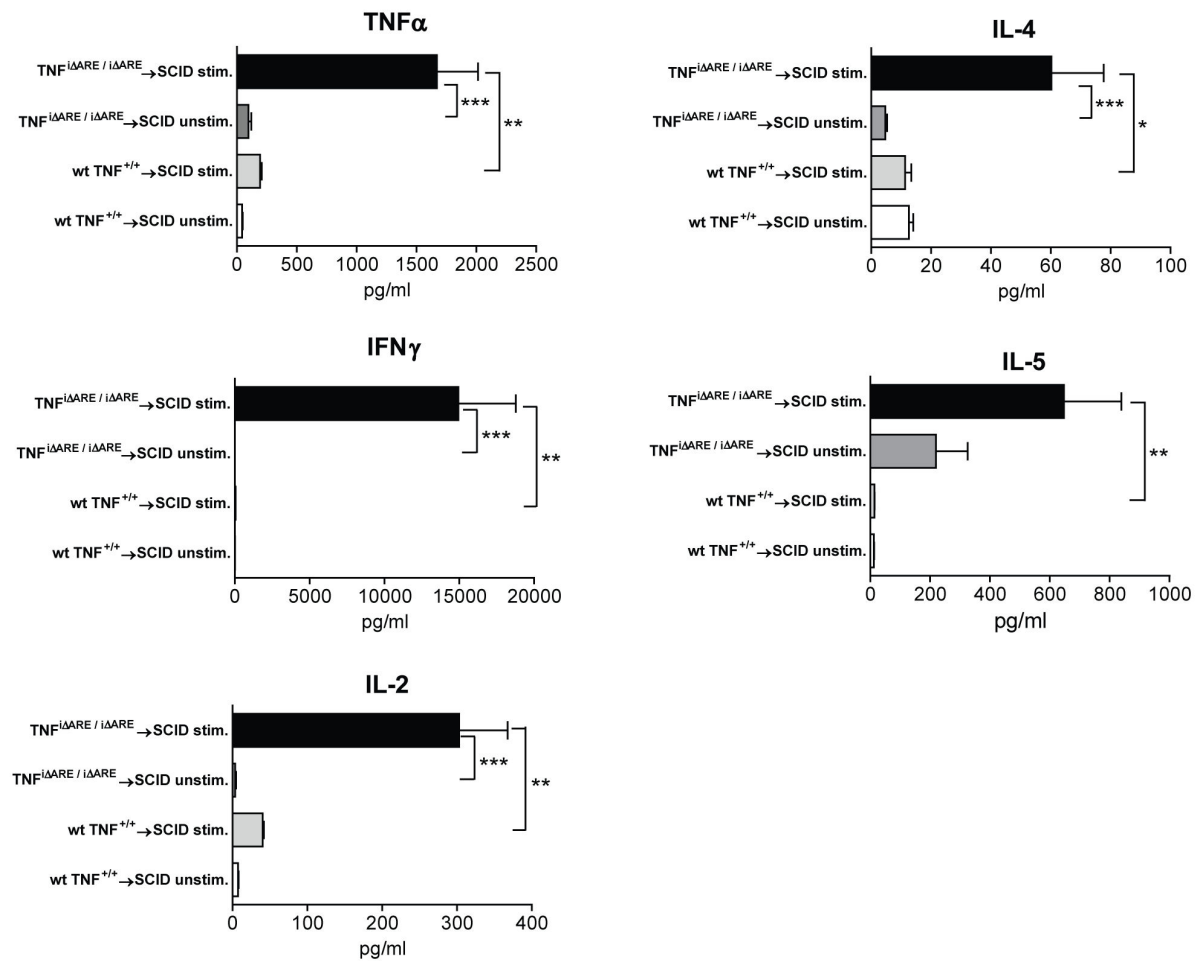
Mucosal effector responses in the adoptive transfer model of TNF^i∆ARE/i∆ARE^→SCID ileitis. CD4^+^ cells from the MLNs of TNF^i∆ARE/i∆ARE^ or wt mice were injected into MHC-matched SCID mice. Recipient mice were euthanized 8 weeks after the transfer. Single cell suspensions from MLNs were cultured in complete medium (10^6^ cells/mL), either with no stimulation or stimulated with immobilized anti-CD3 monoclonal antibody for 48 hours. Concentration of IFNγ, IL-4, IL-5, IL-2, and TNFα were concomitantly determined in culture supernatants by a cytometric bead array. In the absence of stimulation, no substantial cytokine secretion was detected by MLN cells from either strain of mice. Upon stimulation with anti-CD3, there was a significant increase in the secretion of all cytokines in MLN cells from TNF^i∆ARE/i∆ARE^ → SCID mice (n=7), but not from wt → SCID recipients (n=4), indicating the presence of effector lymphocytes in the former, but not the latter, group. Individual mice were processed separately. Three separate experiments were performed, similar to Figure 4. Graphs represent mean values ± SEM for each condition.

Finally, we sought to phenotypically characterize the effector response induced by TNF^i∆ARE/i∆ARE^ CD4^+^ lymphocytes upon adoptive transfer into SCID recipients by measuring the cytokine production profile by stimulated MLN cells from the recipient SCID mice. Our results showed that the induction of ileitis was associated with the presence of both Th1- and Th2-type effector responses, since we observed increased secretion of IFNγ, as well as IL-4 and IL-5 (Figure 5). In particular, upon stimulation with anti-CD3, MLN cells from SCID mice receiving TNF^i∆ARE/i∆ARE^ CD4^+^ cells secreted higher amounts of IFNγ than those receiving wt CD4^+^ cells (TNF^i∆ARE/i∆ARE^ → SCID = 14,950 ± 3,835 pg/ml vs. wt → SCID mice = 57.9 vs. 3.3 pg/ml, P<0.01). In addition, secretion of IL-2 and TNFα were also higher (IL-2: TNF^i∆ARE/i∆ARE^ → SCID = 303 ± 64.8 pg/ml vs. wt → SCID mice = 40.4 ± 1.7 pg/ml, P<0.01; TNFα: TNF^i∆ARE/i∆ARE^ → SCID = 1,672 ± 343 pg/ml vs. wt → SCID mice = 194 ± 13.5 pg/ml, P<0.01). In regard to Th2-type cytokines, the secretion of IL-4 was generally low, although significantly higher in SCID mice receiving cells from iTNF^ΔARE/ΔARE^ compared to wt mice (IL-4: TNF^i∆ARE/i∆ARE^ → SCID = 60.2 ± 17.5 pg/ml vs. wt → SCID mice = 11.3 ± 2.1 pg/ml, P<0.05). Conversely, secretion of IL-5, a pivotal effector Th2 cytokine, was highly elevated in TNF^i∆ARE/i∆ARE^ → SCID mice (TNF^i∆ARE/i∆ARE^ → SCID = 647 ± 192 pg/ml vs. wt → SCID mice = 13.6 ± 0.4 pg/ml, P<0.01). In all, these results indicate that the MLNs in TNF^i∆ARE/i∆ARE^ mice are populated with effector CD4^+^ lymphocytes that possess the ability to induce ileitis upon adoptive transfer to immunologically naïve mice. Based on our analysis of cytokine profiles in recipient mice, the adoptively transferred ileitis appears to be mediated by a mixed Th1/Th2 immunological response.

## Discussion

In the present study, we report the histopathological and immunological characteristics of the TNF^i∆ARE/i∆ARE^ mouse strain, a novel murine model of chronic small intestinal inflammation. We demonstrate that intestinal-specific overexpression of TNFα is sufficient for the induction of severe ileitis, but not for the development of arthritis, which differentiates TNF^i∆ARE/i∆ARE^ mice from the well-characterized TNF^ΔARE/+^ strain that overexpresses TNFα systemically and develops chronic ileitis and inflammatory arthritis. We show that intestinal epithelial-derived TNFα overproduction leads to enrichment of the mucosal immune system by activated lymphocytes and disruption of normal apoptotic pathways. Finally, we provide evidence that these activated lymphocytes display properties of CD4^+^ effector cells, as they are capable of inducing ileitis in immunologically naïve recipients via the generation of a mixed Th1/Th2 cytokine response.

In recent years, evidence has accumulated to support a central role for the intestinal epithelium in the pathogenesis of IBD [18]. Possible mechanisms that could lead to disease include defective barrier function, recruitment of neutrophils to the mucosa, or immunomodulatory dysfunction. In the present study, we provide evidence for an important role of the intestinal epithelium in IBD pathogenesis—namely, a mucosal source of localized TNFα overproduction. In the TNF^i∆ARE/i∆ARE^ mouse, intestinal epithelial-specific overexpression of TNFα is sufficient to induce the development of severe ileitis.

Several lines of evidence support IECs as a local source of TNFα overproduction. Exposure of IEC lines to invasive bacteria results in production of secreted TNFα [19]. In addition, TNFα mRNA transcripts can be detected in Paneth cells under normal conditions, and are expressed at much higher levels by these cells in patients with necrotizing enterocolitis [20]. Our results are consistent with a recent report by Roulis et al. showing that intestinal-specific overexpression of TNFα under the control of a Villin Cre intestinal-specific promoter also generates chronic ileitis with similar features in TNF^∆ARE^ mice [21]. However, our study uses a different intestinal-specific promoter and adoptive transfer of specific lymphocyte populations. Altogether, these results strongly support that IEC-derived TNFα overproduction also occurs in patients with IBD.

Epithelial-derived overproduction of TNFα could lead to the development of chronic intestinal inflammation through multiple possible mechanisms. For example, the severely impaired villous architecture seen in TNF^i∆ARE/i∆ARE^ mice may cause defective intestinal barrier function and enhanced epithelial permeability. In fact, TNFα-mediated injury to the intestinal barrier has been previously linked to the induction of epithelial cell apoptosis [22,23]. Blockade of such apoptotic pathways is central to the beneficial effects of anti-TNFα antibodies in treating Crohn’s-like murine ileitis in the SAMP1/YitFc mouse model of CD [8]. In addition, these data support human studies showing restoration of the epithelial barrier in patients with IBD following successful treatment with anti-TNFα drugs [24].

An alternative mechanism for the development of TNF^i∆ARE/i∆ARE^ ileitis is that IECs contribute to shaping immune responses within the gut mucosa, in addition to their tradition role in maintaining barrier function. This concept is supported by our finding that the lymphocytic compartment was expanded in the MLNs and LP of TNF^i∆ARE/i∆ARE^ mice, as reflected by the significantly higher total inflammatory index. This effect was not attributable to increased expression of adhesion molecules, as we were unable to detect any significant differences in the expression of α_4_, α_M_, or β_7_ integrins on CD4^+^ cells between TNF^i∆ARE/i∆ARE^ and wt mice (data not shown). In addition, within this expanded lymphocytic population, we detected elevated numbers of CD4^+^ cells with an activated phenotype (i.e. expressing surface CD69, CD25, or lacking CD62L expression). Moreover, these cells displayed properties of effector lymphocytes, as they were capable of transferring ileitis to immunologically naïve recipients. In all, our data demonstrate that IECs, when overexpressing TNFα, become capable of inducing a pro-inflammatory mucosal immune response through the generation of effector CD4^+^ lymphocytes.

In support of our findings, recent work in humans showed that IECs from IBD patients induce CD4^+^ cell proliferation and cytokine secretion in a MHC-II dependent manner [25]. Interestingly, we observed no strict polarization of the epithelial-induced CD4^+^ effector cell population in TNF^i∆ARE/i∆ARE^ mice, as they were capable of secreting both Th1 and Th2 cytokines. Similar mixed immunophenotypes have been frequently reported during experimental inflammation, such as in spontaneous SAMP1/YitFc ileitis or IL-10^-/-^ colitis [26,27]. Furthermore, patterns of combined immune responses also occur in subsets of patients with IBD and may be implicated in different phases of disease development [27].

Recently, the role of TNFα in mucosal immunity has been reconsidered. Once believed to be of pure proinflammatory nature, this pivotal cytokine has been shown recently to exert protective effects during homeostatic conditions. In fact, mice deficient in TNFα or TNFRI-signaling are more susceptible to DSS-induced colitis [28,29]. In addition, the beneficial effects of probiotics may be mediated through the early upregulation, rather than suppression, of pro-inflammatory mucosal responses (including TNFα) [30]. In light of this new hypothesis, the development of ileitis in TNF^i∆ARE/i∆ARE^ mice in the presence of high levels of mucosal TNFα may be explained in various ways. On the one hand, due to their abnormal genetic machinery, epithelial cells from these mice may secrete excessive amounts of TNFα in response to physiologic stimuli. Interestingly, severe histological disease was present in homozygous (TNF^i∆ARE/i∆ARE^), but not heterozygous (TNF^i∆ARE/+^) mice, despite elevated local and systemic amounts of TNFα protein in the latter. Therefore, we speculate that a threshold for mucosal TNFα concentration exists, which must be exceeded before proinflammatory responses are triggered and intestinal inflammation develops. On the other hand, deletion of AU-rich elements may render the *tnf* gene unresponsive to regulatory signals induced by cytokines such as IL-10. Consequently, once started, TNFα-mediated, proinflammatory responses may continue unopposed and result in chronic intestinal inflammation. The clinical relevance of such a pathway has been recently confirmed in patients carrying a mutation in the *IL-10R* gene; these individuals exhibit defective inhibition of LPS-induced TNFα along with the early development of IBD [31]. Finally, dysregulation of apoptotic pathways may also be involved in the pathogenesis of TNF^i∆ARE/i∆ARE^ ileitis. In our study, a large expansion of MLN lymphocytes expressing surface FasL was observed in TNF^i∆ARE/i∆ARE^ mice. This would indicate an increase in apoptosis, given the uniform expression of Fas on MLN cells. However, this does not appear to be the case, as it contrasts with the vast expansion of mucosal lymphocytes in TNF^i∆ARE/i∆ARE^ mice. Defective Fas/FasL association and/or impaired downstream signaling may explain these contradictory findings [8].

The pathogenic relationship between extraintestinal manifestations and intestinal inflammation in IBD is poorly understood, partly because very few experimental models of intestinal inflammation display extraintestinal disease, (a small percentage of SAMP1/YitFc mice, which develop spontaneous Crohn’s-like ileitis with 100% penetrance, also develop perianal disease, and the HLA-B27 transgenic colitic rat displays arthritis) [32,33]. When examined comparatively with its closely related TNF^∆ARE/+^ strain that systemically overproduces TNFα and develops chronic ileitis and arthritis, the TNF^i∆ARE/i∆ARE^ strain offers a unique opportunity to study common and diverse pathways in joint and intestinal pathologies. Interestingly, the two models showed comparable circulating levels of TNFα. Nevertheless, this systemic increase was not sufficient for the development of arthritis in TNF^i∆ARE/i∆ARE^ mice. Presumably, local secretion of TNFα by synoviocytes is a critical factor for the generation of proinflammatory conditions that lead to joint destruction in the TNF^ΔARE/+^ mice; mucosal overexpression is present in both models and leads to the common phenotype of ileitis. Interestingly, low-grade inflammation is observed in the colon of TNF^i∆ARE/i∆ARE^ mice, in accordance with the minimal expression of the I-FABP promoter in colonocytes. Taken together, these results support the concept that the local cytokine microenvironment is the decisive factor for development of a particular inflammatory phenotype.

In conclusion, we demonstrate in the present study that intestinal epithelial-derived TNFα in TNF^i∆ARE/i∆ARE^ mice induces pro-inflammatory innate and adaptive mucosal immune responses, resulting in chronic intestinal inflammation. This murine model displays novel immunological characteristics and offers a useful tool for dissection of the pathways that underlie TNF-mediated intestinal pathology, as well as the pathogenic relationship between IBD and its associated extraintestinal manifestations.
